# Retraction: Novel Mechanism of Inhibition of Dendritic Cells Maturation by Mesenchymal Stem Cells via Interleukin-10 and the JAK1/STAT3 Signaling Pathway

**DOI:** 10.1371/journal.pone.0194455

**Published:** 2018-03-14

**Authors:** Wen-hua Liu, Jing-jin Liu, Jian Wu, Lu-lu Zhang, Fang Liu, Li Yin, Mao-mao Zhang, Bo Yu, and the PLOS ONE Editors

Following publication of this article [1], concerns were raised regarding the presented data.

In Figure 5, the P-JAK1 and STAT3 Western blot panels are duplicates.

Four pairs of panels are duplicated in Figure 7:

7A panels CD86 and OX62.
7B panels CD86 and OX62.
7A panel CD11b/c and 7C panel CD11b/c.
7A panel MHC-II and 7B panel CD80.

Three pairs of panels are duplicated between Figure 7 and Figure 2:

7B panel CD86 and 2A panel CD86.
7D panel CD11b/c and 2A panel CD11b/c.
7E panel CD11b/c and 2B panel CD11b/c.

Concerns were raised regarding the accuracy of the percentage data presented for region M2 of the erroneously duplicated FACS plots.

Two pairs of panels are duplicated in Figure 8:

Figure 8A: P-JAK1 and JAK1 Western blot panels.
Figure 8C: STAT3 and P-STAT3 Western blot panels.

Uncropped, raw blot images are no longer available for further evaluation.

In view of the concerns regarding the reliability of the results and the absence of the raw data images, the authors and *PLOS ONE* Editors retract this article. The authors wish to apologize to readers.

WHL, JW, LLZ, LY, MMZ and BY agree with the retraction. JJL, and FL could not be reached.
